# White matter microstructure and local coherence of functional MRI in major depression

**DOI:** 10.1192/j.eurpsy.2023.1163

**Published:** 2023-07-19

**Authors:** S. Breit, N. Denier, N. Mertse, S. Walther, A. Federspiel, R. Wiest, T. Bracht

**Affiliations:** 1 University Hospital of Psychiatry and Psychotherapy, University of Bern; 2 Translational Research Center, University Hospital of Psychiatry and Psychotherapy, University of Bern; 3 Institute of Diagnostic and Interventional Neuroradiology, University of Bern, Bern, Switzerland

## Abstract

**Introduction:**

Anhedonia is a loss of pleasure and interest in activities and a core symptom of major depressive disorder (MDD). Diffusion tensor imaging studies show evidence for white matter (WM) alterations in the superior longitudinal fasciculus (SLF) of patients with MDD, already in the early stage of illness. SLF fibers extend from the parietal lobe to prefrontal regions that are important for attention, motivation, decision-making and reward processing.

**Objectives:**

The present study focuses on the relationship between WM-integrity and anhedonia in patients with MDD. We hypothesize that WM-alterations are present in the SLF of depressed patients with motivational anhedonia.

**Methods:**

Thirty-nine patients with MDD and 19 healthy controls matched for age and gender underwent diffusion-weighted magnetic resonance imaging. Voxel-wise statistical analysis of fractional anisotropy (FA) data was performed using FSL-Tract-Based Spatial Statistics (TBSS) software. Whole brain voxel-wise comparison in local coherence (LCOR), a measurement of resting state fMRI connectivity strength between a given voxel and the neighbouring areas in the brain, were compared between patients and healthy controls. We used the sum value of item 1 and 7 of the Hamilton rating Scale for depression (HAM-D) and the CORE non-interactiveness value to assess motivational anhedonia.

**Results:**

TBSS-analyses revealed reduced FA in the left SLF of depressed patients and we found a correlation with motivational anhedonia and LCOR in temporo-parietal regions of depressed patients.

**Conclusions:**

These findings suggest that WM-alterations in the SLF might be associated with motivational aspects of anhedonia and predict motivation of reward in MDD.

**Disclosure of Interest:**

None Declared

